# The long noncoding RNA TARID regulates the CXCL3/ERK/MAPK pathway in trophoblasts and is associated with preeclampsia

**DOI:** 10.1186/s12958-022-01036-8

**Published:** 2022-11-19

**Authors:** Lingyun Liao, Min Liu, Yijie Gao, Xiaohong Wei, Yangxue Yin, Linbo Gao, Rong Zhou

**Affiliations:** 1 Department of Obstetrics and Gynecology, West China Second University Hospital, Sichuan University, Key Laboratory of Birth Defects and Related Diseases of Women and Children (Sichuan University) of Ministry of Education, Chengdu, Sichuan China; 2 Center for Translational Medicine, Key Laboratory of Birth Defects and Related Diseases of Women and Children (Sichuan University), Ministry of Education, Department of Obstetrics and Gynecology, West China Second University Hospital, Sichuan University, Chengdu, Sichuan China

**Keywords:** lncRNA, Preeclampsia, TARID, CXCL3, ERK/MAPK

## Abstract

**Background:**

The widely accepted explanation of preeclampsia (PE) pathogenesis is insufficient trophoblast invasion and impaired uterine spiral artery remodeling. However, the underlying molecular mechanism remains unclear.

**Methods:**

We performed transcriptome sequencing on placentas of normal and PE patients and identified 976 differentially expressed long noncoding RNAs (lncRNAs). TCF21 antisense RNA inducing demethylation (TARID) was one of the most significantly differentially expressed lncRNAs and was negatively correlated with the systolic and diastolic blood pressure in PE patients. Furthermore, we verified the effect of TARID on the biological behavior of trophoblasts and performed UID mRNA-seq to identify the effectors downstream of TARID. Then, co-transfection experiments were used to better illustrate the interaction between TARID and its downstream effector.

**Results:**

We concluded that the downregulation of TARID expression may inhibit trophoblast infiltration and spiral artery remodeling through inhibition of cell migration, invasion, and tube formation mediated through the CXCL3/ERK/MAPK pathway.

**Conclusions:**

Overall, these findings suggested that TARID may be a therapeutic target for PE through the CXCL3/ERK/MAPK pathway.

**Supplementary Information:**

The online version contains supplementary material available at 10.1186/s12958-022-01036-8.

## Background

The incidence of preeclampsia (PE) in all pregnancies is approximately 2 to 8% [1, 2]. There is no effective treatment for PE except termination of pregnancy. The widely accepted “two-stage theory” suggests that the infiltrative capacity of extravillous trophoblastic cells in PE is impaired, resulting in extremely inadequate placental implantation and uterine spiral arterial remodeling, which in turn leads to decreased placental perfusion and a series of signs and symptoms of PE [3, 4]. However, the mechanisms responsible for insufficient trophoblast infiltration and impaired spiral arterial remodeling remain to be studied.

A growing number of studies have utilized transcriptome sequencing to determine the roles played by long noncoding RNAs (lncRNAs) in the pathogenesis of multiple diseases. LncRNAs, with a length of at least 200 nt, play indispensable roles in complex biological functions, including post-transcriptional regulation, organization of protein complexes, cell–cell signaling, and allosteric regulation of proteins [5–10]. A few studies have shown that lncRNAs are involved in PE regulation by altering the biological behavior of trophoblasts. For example, SH3PXD2A-AS1 recruited CCCTC-binding factor to the promoter of SH3PXD2A to restrain cell invasion and migration [5]. INHBA-AS1 inhibited the invasion and migration of trophoblasts by binding to the promoter of TNF receptor-associated factor 1, inhibiting transcription factor CENPB [6]. MEG3 promoted the proliferative and invasive capacities of trophoblasts by activating the RAS-MAPK pathway [8]. Although an increasing number of recent studies have been performed to determine the expression differences of lncRNAs in PE and in normal pregnancy, due to the large number and complex function of lncRNAs, it is obvious much work remains to be done, especially mechanistic analyses.

We recently carried out transcriptome sequencing on placentas from five pairs of pregnant women with normal pregnancies or PE with matching gestational ages and identified 976 differentially expressed lncRNAs. TARID was among the most differentially expressed lncRNAs. Here, we report our identification of TARID as a potential causal factor of PE and the TARID downstream pathways involved in PE.

## Methods

### Clinical features and placenta preparation

We enrolled patients according to the American College of Obstetricians and Gynecologists (ACOG) diagnostic criteria for PE [11]. Placenta samples were collected from women with PE (*n* = 43) and normal pregnancies (*n* = 40) during cesarean section at West China Second Hospital of Sichuan University between November 2019 and June 2021. Samples were taken within 30 minutes placenta isolation. Specifically, 1 cm^3^ of surface parent villous tissues containing a large number of trophoblasts were obtained, and calcification and blood vessels were avoided during collection. Samples were washed with ice phosphate buffered saline (PBS) to prevent the interference of blood cells, and immediately stored in a -150 °C refrigerator. This study was approved by the Ethics Committee of West China Second Hospital (No. MRER-2019-032), and all participants in the study signed informed consent forms.

### Transcriptome sequencing

Five pairs of placentas in women with normal pregnancy or PE matched by gestational age were selected for transcriptome sequencing (Oebiotech, China). The clinical information of the five selected pairs of pregnant women is listed in Supplementary Table 1 (Additional file 1).

### RNA extraction and quantitative real-time polymerase chain reaction (qRT–PCR)

Total RNA was extracted with an RNA extraction kit (Bioteke, China), and the concentration of RNA was measured with an ultramicro spectrophotometer (Implen, Germany). Then, the PrimeScriptTM RT reagent kit (TaKaRa, Japan) was used for reverse transcription according to the manufacturer’s protocols. qRT–PCR was carried out using ChamQ Universal SYBR qPCR Master Mix (Vazyme, China). All experiments were conducted in triplicate. The relative expression levels were analyzed using the 2^−ΔΔCt^ method, and β-actin was the internal control. The primers for the qRT–PCRs are shown in Supplementary Table 2 (Additional file 1).

### Cell culture and transfection

The HTR8/SVneo cell line (National Collection of Authenticated Cell Cultures, China) and JAR cell line (Shanghai Zhong Qiao Xin Zhou Biotechnology, China) were cultured in RPMI 1640 medium (HyClone, USA) supplemented with 10% fetal bovine serum (Gibco, USA) and 1% penicillin–streptomycin (Gibco) in humidified air at 37 °C with 5% CO_2_.

An TARID overexpression pcDNA3.1(+) vector (pcDNA3.1-TARID) (BGI, China), TARID antisense oligonucleotide (ASO-TARID) (RiboBio, China), CXCL3 overexpression pcDNA3.1(+) vector (pcDNA3.1-CXCL3) (Tsingke, China), and small-interfering RNA against CXCL3 (si-CXCL3) (RiboBio, China) were transfected into cells using Lipofectamine™ 3000 transfection reagent (Invitrogen, USA) when cell confluency was 50–70% in six-well plates. Co-transfection of pcDNA3.1-TARID and si-CXCL3 or ASO-TARID or pcDNA3.1-CXCL3 was performed to study the mechanism of TARID effects downstream. The assays were carried out from 24 to 48 h after transfection.

### Fluorescence in situ hybridization (FISH)

TARID, U6 or 18S probes (RiboBio, China) were analyzed to detect the localization and distribution of cells with a FIISH kit (RiboBio, China). First, placental paraffin sections or cell slides were prepared. Then, 2.5 μL of 20 μM probe was added and incubated overnight at 37 °C. The next day, the slides were rinsed and then incubated with Cytokeratin 7 rabbit monoclonal antibody (mAb) (1:200, ABclonal, China) overnight at 4 °C. On the third day, the cells were incubated with a fluorescent secondary antibody and then counterstained with 40′,6-diamidino-2-phenylindole (DAPI). Images were taken with a laser scanning confocal microscope (Olympus, Japan).

### Subcellular fractionation

Nuclear and cytosolic fractions were prepared from HTR8/SVneo cells with a PARIS™ Kit (Life Technologies, USA). qPCR assays were performed to detect TARID, U6, and β-actin in the nucleus and cytoplasm of the cells. The subcellular expression levels of TARID, U6, and β-actin are expressed as relative percentages. U6 and β-actin were included as nuclear and cytosolic markers, respectively.

### Transwell assays

A total of 5 × 10^4^ HTR8/SVneo cells or 1 × 10^5^ JAR-transfected cells were seeded in the upper compartment of a Transwell insert with an aperture of 8 μm for the cell migration assay. HTR8/SVneo cells (1 × 10^5^) or JAR-transfected cells (2 × 10^5^) were seeded on upper Matrigel-coated compartment for the cell invasion assay. After incubation for 24 h, 4% formaldehyde was used for fixation for 30 minutes, and crystal violet was added for 4 minutes. Then, the cells were rinsed with double distilled water, the cells on the top of the membrane were gently wiped with cotton swabs, and pictures were taken under the microscope. All experiments were conducted in triplicate.

### Cell viability assay

The effect of TARID on trophoblast proliferation was measured by cell counting kit-8 (CCK-8) (Saint-Bio, China) assay. HTR8/SVneo- or JAR-transfected cells were seeded in 96-well plates at 4000 cells per well. The optical density (OD) value was measured at 490 nm with a microplate reader (TECAN, Switzerland) at a predetermined time. The results are representative of three individual experiments.

### Tube formation assay

Angiogenesis slides (Ibidi, Germany) were coated with 10 μL of Matrigel (BD Biosciences, USA), which was allowed to condense at 37 °C for 2 h. A total of 2000 transfected HTR8/SVneo cells were plated on the top layer. After incubation for 4 h at 37 °C, images were acquired for tube formation analysis. All experiments were conducted in triplicate.

### Flow cytometry for apoptosis

For an apoptosis analysis, the cells were stained with Annexin V-fluorescein isothiocyanate (FITC) and propidium iodide (PI) (KeyGen, China). Harvested cells were analyzed by flow cytometry (Millipore, USA) according to the manufacturer’s recommendations. Three individual experiments were performed.

### UID mRNA-seq

Three groups of HTR8/SVneo cells transfected with pcDNA3.1-TARID or pcDNA3.1-NC were selected to extract RNA, and second-generation sequencing of UID mRNA was then performed (SEQHEALTH, China).

### Western blot analysis

Proteins from placentas in women with normal pregnancy or PE (matched for gestational age, fetal sex and primiparity) and cell lines were extracted using a total protein extraction kit (Beyotime, China). The protein concentration was determined using a BCA protein quantification kit (Yeasen, China). All proteins were standardized to a concentration of 3 μg/μL and denatured at 100 °C for 5 minutes in SDS–PAGE protein loading buffer (5X) (Beyotime, China). The proteins were separated with a PAGE gel quick preparation kit (Yeasen, China) and transferred to polyvinylidene difluoride (PVDF) membranes (Millipore, USA). Subsequently, the membranes were blocked with 5% skim milk powder (Solarbio, China) for 1 h and incubated with primary antibodies against CXCL3 (1:500, Sigma, USA), ERK1/2 (1:1000, ABclonal, China), p-ERK1/2 (1:1000, ABclonal, China) or β-actin (1:5000, ABclonal, China) overnight at 4 °C. Next, the membranes were washed three times with TBST and then incubated with a secondary antibody (1:100000, ABclonal, China) for 1 h at room temperature. Finally, SuperKine™ West Femto Maximum Sensitivity Substrate (Abbkine, China) was used to detect the chemiluminescence intensity with a ChemiDoc™ MP Imaging System (Bio-Rad, USA). Image Lab 6.0 software (Bio-Rad, USA) was used for gray value calculation. All experiments were conducted in triplicate.

### Immunofluorescence

Placental tissue from normal pregnancies and patients with preeclampsia were subjected to immunofluorescence staining with CXCL3 Rabbit mAb (1:200, Sigma, USA) and Cytokeratin 7 Rabbit mAb (1:200, ABclonal,China) (Cdllsw, China).

### Statistical analysis

Statistical analyses were performed using SPSS version 26.0.0.0 (IBM, USA) and GraphPad Prism 9.0.1 (GraphPad Software, USA) software. The normality of the data was analyzed by Kolmogorov–Smirnov normality test. Differences between two groups were analyzed by Student’s t test for normally distributed continuous variables and by the Mann–Whitney U test for nonnormally distributed continuous variables. Pearson’s chi-squared test was used for analyzing categorical variables. The correlation between two variables was analyzed by Pearson correlation. Binary logistic regression was used to examine the association between placental TARID or CXCL3 mRNA levels, and the risk of developing PE was determined by calculating unadjusted and adjusted odds ratios (ORs). Body mass index (BMI) and gestational age were included in the analysis following the processes reported in a previous study [12, 13]. A *P* value less than 0.05 was regarded as statistically significant.

## Results

### Clinical characteristics of the study population

The clinical characteristics of PE patients (*n* = 43) and normal controls (*n* = 40) are shown in Table 1. Analysis of the clinical features showed that the PE group had abnormal SBP, DBP, proteinuria, ALT, AST, albumin, and LDH levels, which were consistent with the diagnostic criteria for PE. Moreover, there was a significant difference in gestational age, body mass index (BMI), and primipara between the two groups, but there was no difference in maternal age. In terms of fetal outcomes, birth weight and birth length were lower in the PE group than in the control group. There was no significant difference in fetal sex between the two groups.

**Table 1:** Clinical characteristics of preeclamptic and normal pregnancies

Variable	Control (*n* = 40)	PE (*n* = 43)	*P* value
Maternal age (year)	32.08 ± 3.5	31(29–35)	> 0.05
BMI (kg/m2)	26.14 ± 2.85	29.13 ± 3.54	< 0.01
SBP (mm Hg)	117.3 ± 9.92	154.63 ± 13.52	< 0.01
DBP (mm Hg)	73.9 ± 8.81	98 ± 10.15	< 0.01
Gestational ages (weeks)	38.6 ± 0.82	36.29 ± 2.32	< 0.01
Primipara	51.28%	74.36%	< 0.05
Proteinuria (g/24 h)	0	0.77(0.48–2.82)	< 0.01
Platelet (10^9^/L)	177(158.25–215.75)	160.79 ± 70.59	> 0.05
ALT (U/L)	13.5(10–19)	27(13–77)	< 0.01
AST (U/L)	20(17–23)	31(21–48.5)	< 0.01
Total bilirubin (umol/L)	8.91 ± 2.91	8.1(5.5–10.8)	> 0.05
Albumin (g/L)	37.55 ± 2.39	33.83 ± 5.54	< 0.01
LDH (U/L)	203.5(160–343.25)	333(248–423.5)	< 0.01
BUN (mmol/L)	3.21 ± 0.76	4.7(3.83–6.12)	< 0.01
Serum creatinine (mmol/L)	44.93 ± 6.82	59.93 ± 14.71	< 0.01
Birth weight (g)	3308.25 ± 429.16	2504.05 ± 715.13	< 0.01
Birth length (cm)	49.5 ± 1.57	45.81 ± 4.53	< 0.01
Fetal sex (male)	55.00%	55.32%	> 0.05

Values shown are mean ± standard deviation or median and interquartile range, unpaired student’s t test, Mann-Whitney U test, Wilcoxon Signed Ranks Test, or Pearson’s chi-squared test was used

*BMI* Body mass index, *SBP* Systolic pressure, *DBP* Diastolic pressure, *ALT* Alanine aminotransferase, *AST* Aspartate aminotransferase, *LDH* Lactate dehydrogenase, BUN Blood urea nitrogen

### TARID is downregulated in the PE placenta and related to blood pressure

**Fig. 1:**
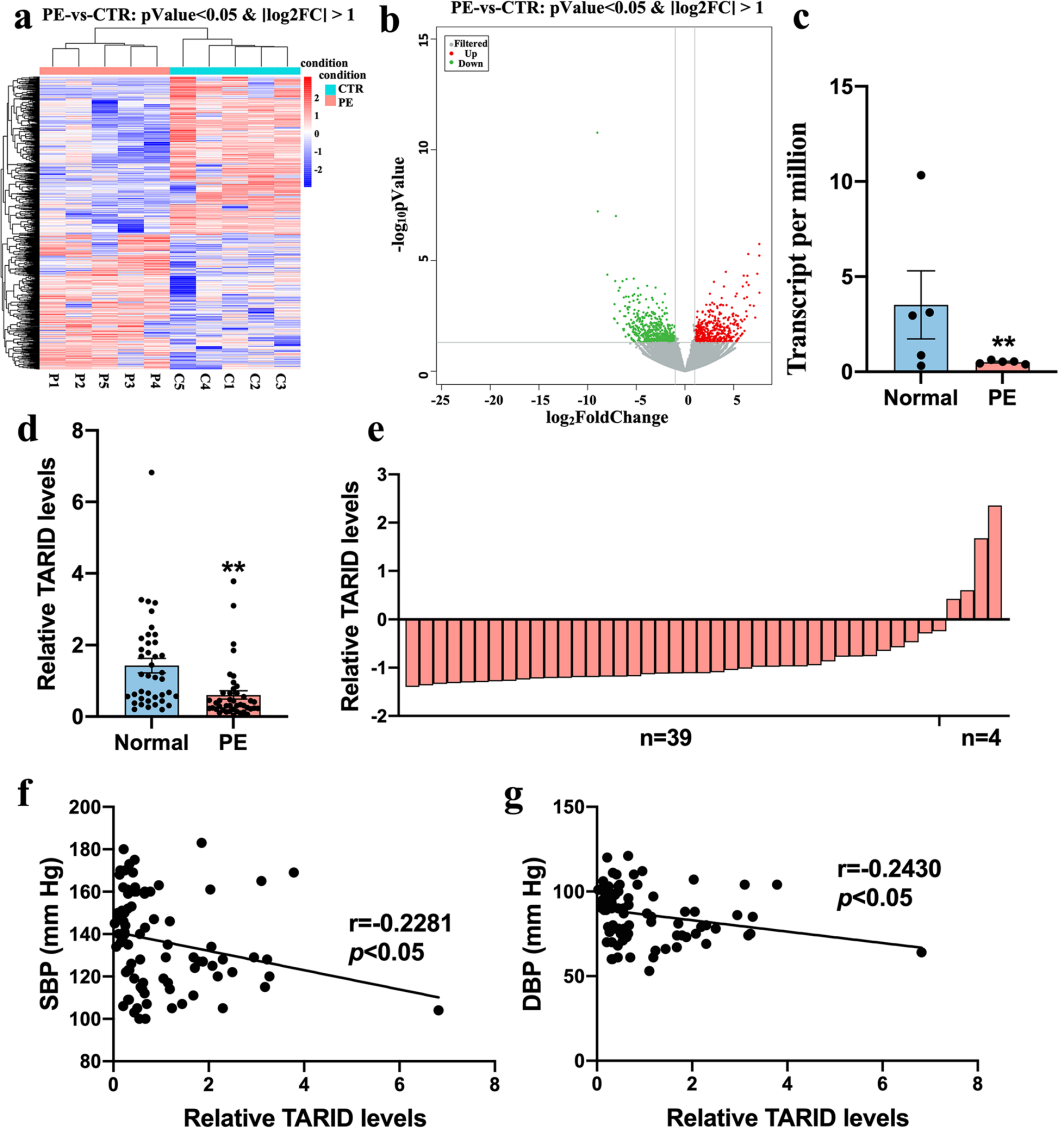
TARID is down regulated in the PE placenta and related to blood pressure. **a** Differential gene grouping cluster diagram. Red indicates the relatively high expression protein coding gene, and blue indicates the relatively low expression protein coding gene. **b** Differentially expressed volcanic map. Gray is the gene with a non-significant difference, and red and green are the genes with a significant difference. **c** TARID expression levels in PE placenta (*n* = 5) compared with normal placentae (*n* = 5) by transcriptome profiling. **d** The relative expression level of TARID was measured by qRT-PCR (Normal = 40, PE = 43). **e** The expression level of TARID is decreased in 91% (39/43) of PE samples compared with the average level of 40 normal samples. The average expression level in normal samples was set as zero. **f** Negative correlation (*r* = − 0.2281) between systolic blood pressure and the relative expression of TARID (Normal = 40, PE = 43) (Pearson correlation analysis). **g** Negative correlation (*r* = − 0.2430) between diastolic blood pressure and the relative expression of TARID (Normal = 40, PE = 43) (Pearson correlation analysis). All experiments were conducted in triplicate; the values are shown as the mean ± SEM; vs. normal, **P < 0.01, *P < 0.05

We carried out transcriptome profiling on 10 placental samples, 5 from PE patients and 5 from normal controls with matching gestational age. We found 976 differentially expressed lncRNAs; the expression of 446 lncRNAs was consistently upregulated, and that of 530 lncRNAs was consistently downregulated (Fig. 1a, b). TARID was among the most differentially expressed lncRNAs, and the expression of TARID was significantly downregulated in the placental transcriptomes of the PE patients (Fig. 1c).

To verify the expression of TARID, we collected placental samples from 43 PE patients and 40 normal subjects and found that TARID was significantly downregulated in 91% (39/43) of the PE patients (Fig. 1d, e). Since the gestational age and BMI were significantly different between the two patient groups in this analysis, binary logistic regression analysis was used for correction. After adjusting for differences in BMI and gestational age, the TARID expression remained significantly lower in the PE group (OR = 0.17) as before correction (OR = 0.14) (Table 2). Moreover, the expression levels of TARID in placentae were negatively correlated with the severity of clinical characteristics, such as systolic (Fig. 1f, *r* = -0.2281) and diastolic blood pressures (Fig. 1g, *r* = -0.2430).

**Table 2:** The unadjusted and adjusted ORs of the relationship between PE and TARID levels

	n	Unadjusted OR (95% CI)	P value	Adjusted OR (95% CI)	P value
Control	40	1	< 0.01	1	< 0.05
Preeclampsia	43	0.14(0.04–0.46)		0.17(0.04–0.79)	

Adjusted for BMI and gestational age; *ORs* Odd ratios, *CIs* Confidence intervals.

### Localization of TARID in the placenta and trophoblasts

**Fig. 2:**
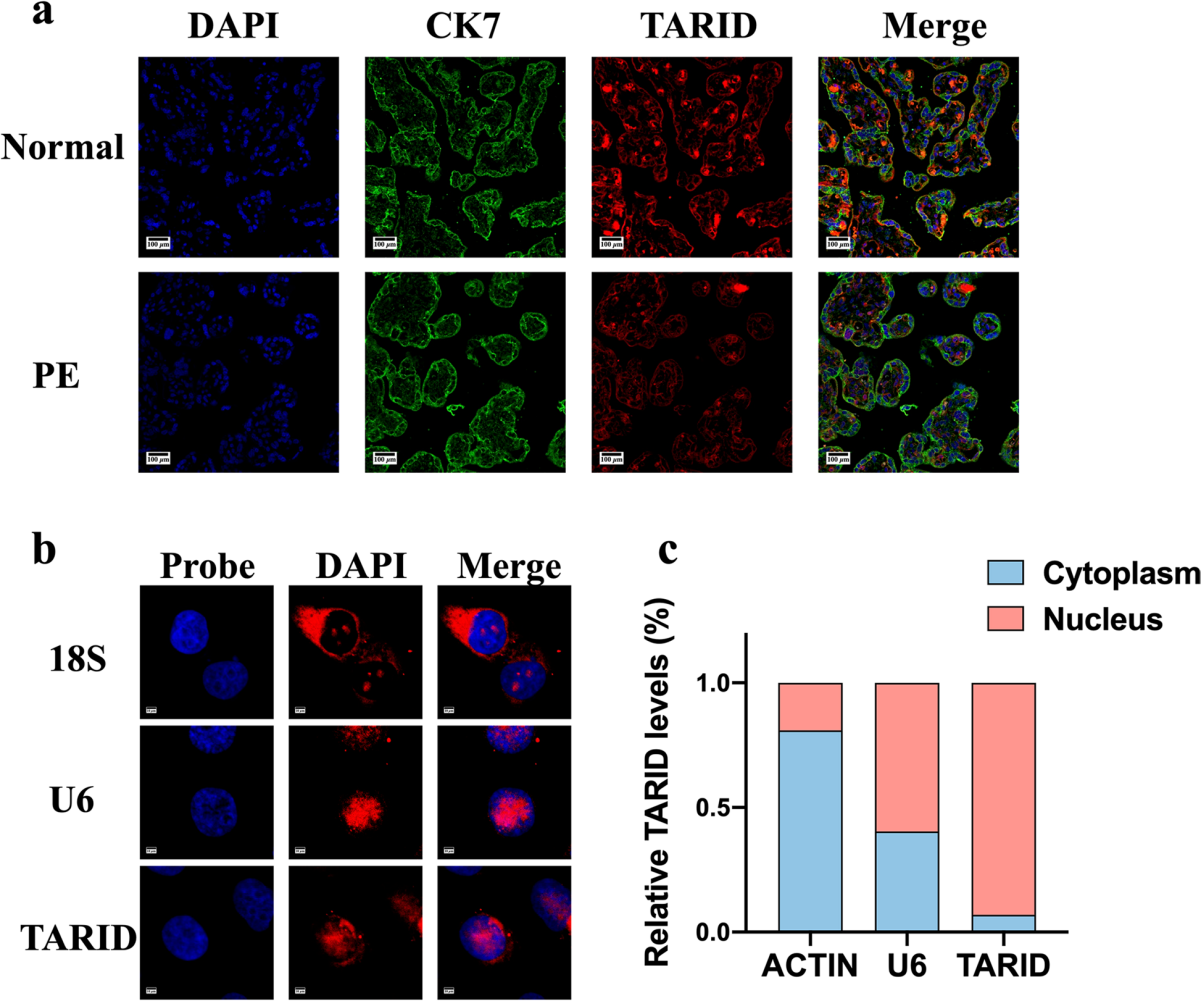
Localization of TARID in placenta and trophoblasts and the transfection efficiency. **a** Representative photomicrographs of TARID fluorescence in situ hybridization (FISH) (400×). **b** The subcellular localization of TARID in HTR8/SVneo cells was detected by FISH assays (2000×). Red, TARID; blue, nucleus. Scale bar, 20 mm. **c** The subcellular localization of TARID in the HTR8/SVneo cells detected by cell fractionation assays. U6, nucleus marker; β-actin, cytoplasm marker. All experiments were conducted in triplicate; the values are shown as the mean ± SEM; vs. vector or ASO，**P < 0.01, *P < 0.05

FISH of the placenta samples revealed the downregulation of TARID expression in the PE placentas and showed that TARID was predominantly expressed in villous trophoblasts (Fig. 2a). In addition, we used cell fractionation and FISH to determine TARID distribution in the cell. The results indicated that TARID was primarily localized in the nucleus (Fig. 2b, c).

### TARID affected the migration and invasion of trophoblasts

**Fig. 3:**
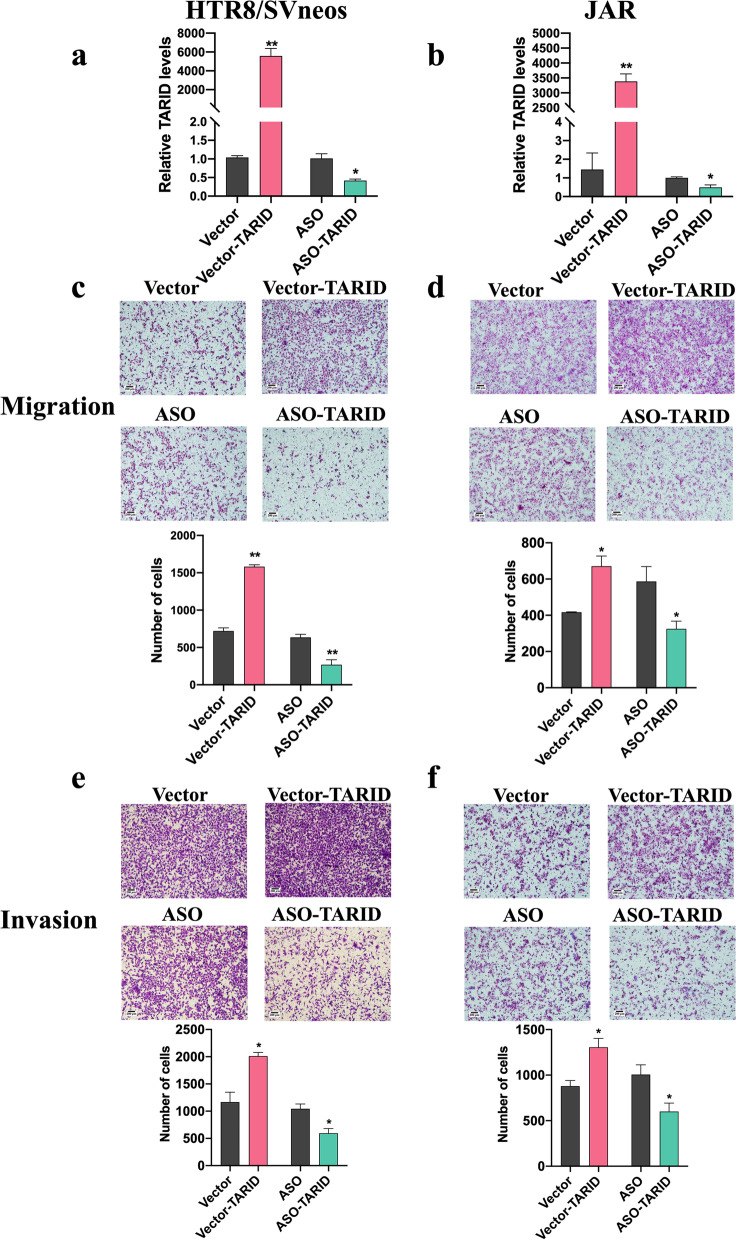
TARID affected migration and invasion of trophoblasts. **a, b** Results of real-time quantitative PCR showing the expression of TARID in HTR8/SVneo (a) and JAR (b) cells transiently transfected with vector-TARID or vector-NC and ASO-TARID or ASO-NC. **c, d** Migration was detected by transwell assay in HTR8/SVneo (c) JAR (d) cells after TARID overexpression and knockdown (100×). Upper panel: photos of cells stained with crystal violet; lower panel: statistics of cells. **e**, **f** Invasion was detected by transwell assay in HTR8/SVneo (e) JAR (f) cells after TARID overexpression and knockdown (100×). Upper panel: photos of cells stained with crystal violet; lower panel: statistics of cells. All experiments were conducted in triplicate; the values are shown as the mean ± SEM; vs. vector or ASO，**P < 0.01, *P < 0.05

Trophoblast invasion and migration are crucial for placental formation, and an imbalance in these parameters is considered to play a key role in the pathogenesis of PE [14]. First, we efficiently overexpressed and knocked down the expression of TARID in HTR8/SVneo and JAR cells (Fig. 3a, b). The Transwell experiments showed that overexpression of TARID significantly increased the migratory and invasive abilities of the HTR8/SVneo cells (Fig. 3c, d), while knocking down TARID expression reduced the migratory and invasive abilities of HTR8/SVneo cells compared with those of the controls (Fig. 3a, b). In addition, we observed the same trend in JAR cells (Fig. 3c, d). These experiments showed that TARID expression can affect the migration and invasion of trophoblasts, thereby affecting the occurrence of PE.

### TARID promoted tube formation of trophoblasts but was not involved in their proliferation or apoptosis

**Fig. 4:**
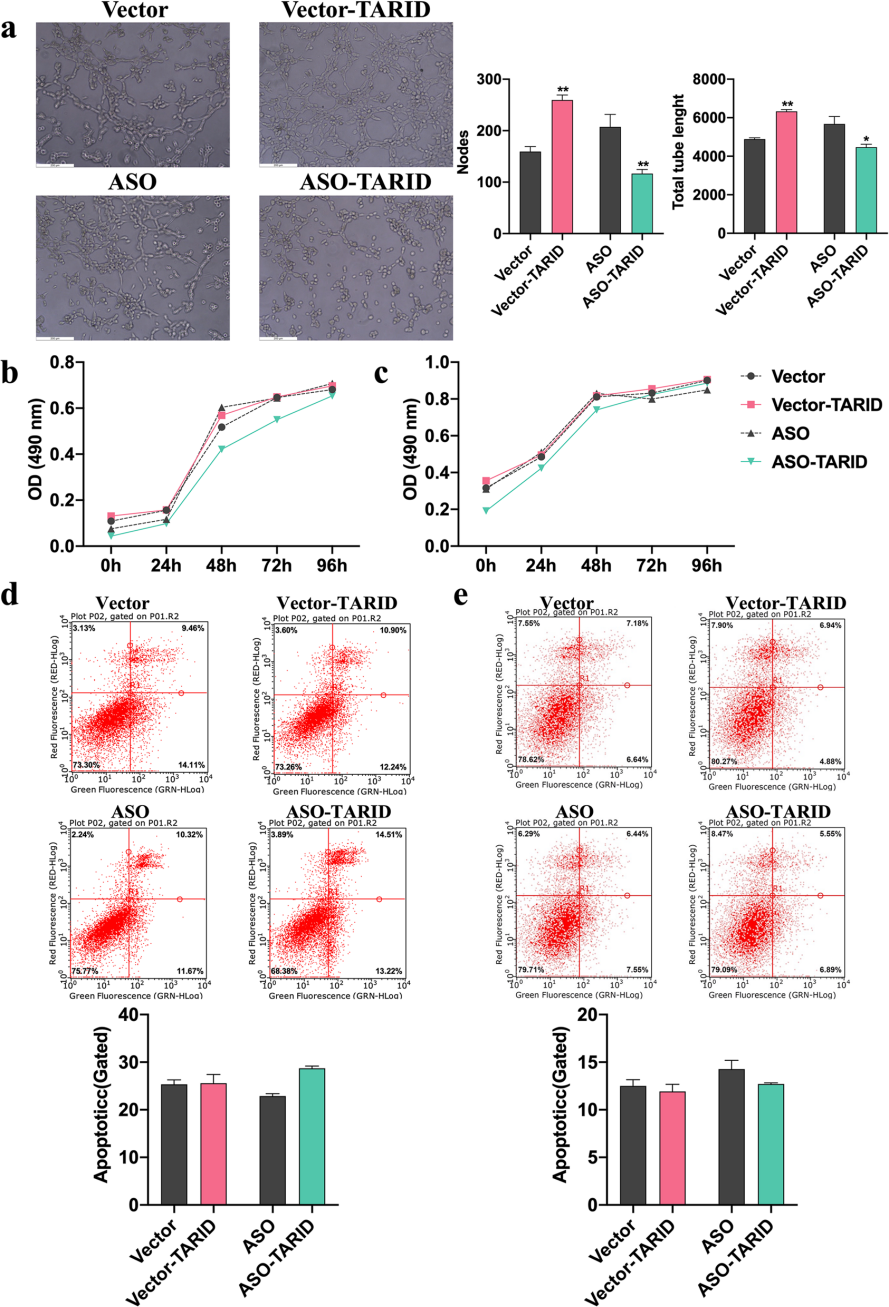
TARID promoted tube formation in trophoblasts but was not involved in proliferation and apoptosis. **a** Tube formation was detected in HTR8/SVneo cells after TARID overexpression and knockdown (400×). Left panel: photos of tube formation; right panel: statistics of cells. **b, c** Cell Counting Kit-8 was used to evaluate the cell proliferation of HTR8/SVneo (b) JAR (c) cells after TARID overexpression and knockdown. **d, e** Apoptosis analysis using flow cytometry on HTR8/SVneo (d) and JAR (e) cells after TARID overexpression and knockdown. Upper panels: histograms of apoptotic cells; lower panels: statistics of apoptotic cells. All experiments were conducted in triplicate; the values are shown as the mean ± SEM; vs. vector or ASO，**P < 0.01, *P < 0.05

Impaired tube formation is one of the underlying mechanisms of PE [15, 16]. Tube formation assays demonstrated that overexpression of TARID significantly increased the nodes and total tube length per field, and downregulation of TARID expression resulted in decreased tube formation of HTR8/SVneo cells (Fig. 4a).

We also performed a by CCK-8 assay to evaluate the effect of TARID on proliferation and found that overexpression or knocked down expression of TARID did not affect the proliferation of the HTR8/SVneo (Fig. 4b) and JAR cells (Fig. 4c). In addition, there were no significant differences in the apoptosis rate between HTR8/SVneo (Fig. 4d) and JAR cells (Fig. 4e).

### CXCL3 is a downstream effector of TARID

**Fig. 5:**
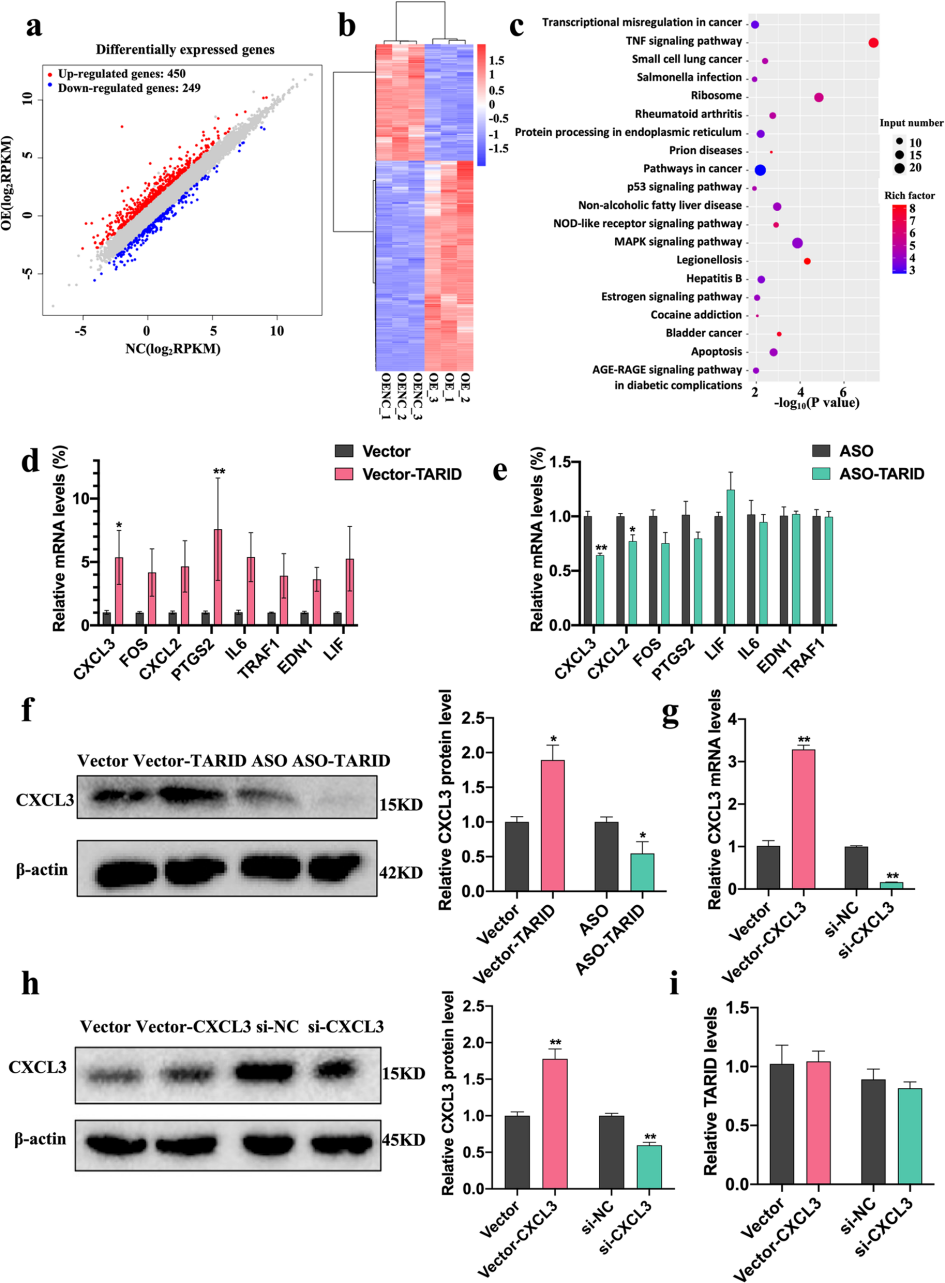
CXCL3 as a downstream effector of TARID. **a** Scatter plot comparing expression levels between groups. **b** Differential gene cluster map. **c** KEGG metabolic pathway map of up-regulated genes. **d, e** The transcription level of TARID target genes in HTR8/SVneo cells with TARID overexpression (d) and knockdown (e). **f** Western blotting results for CXCL3 in HTR8/SVneo cells with TARID overexpression and knockdown. **g, h** Results of real-time quantitative PCR (g) and western blotting (h) showing the expression of CXCL3 in HTR8/Svneo cells transiently transfected with vector-CXCL3 or vector and si-CXCL3 or si-NC. **i** Results of real-time quantitative PCR showing the expression of TARID in HTR8/Svneo cells transiently transfected with vector-CXCL3 or vector and si-CXCL3 or si-NC. All experiments were conducted in triplicate; the values are shown as the mean ± SEM; vs. vector or ASO，**P < 0.01, *P < 0.05

To explore the possible downstream effectors of TARID that affect cell migration, invasion, and tube formation, In HTR8/SVneo cells, TARID was overexpressed, and then, RNA sequencing was performed. The expression of 450 RNAs was increased, and that of 249 RNAs was decreased (Fig. 5a, b). Then, Gene Ontology (GO) and Kyoto Encyclopedia of Genes and Genomes (KEGG) analyses were performed and showed that the TNF signaling pathway was significantly enriched in genes with increased expression (Fig. 5c). Next, we verified the 8 most differentially expressed genes (sorted by log fold change (logFC)) and the pathways in which they were enriched, and CXCL3 was the most significantly differentially expressed mRNA and protein in the cells with either TARID overexpression or expression knockdown (Fig. 5d-f). These results were consistent with the sequencing results. To further investigate the regulatory relationship between TARID and CXCL3, we constructed CXCL3-overexpressing and CXCL3-knockdown cells (Fig. 5g, h). We found no significant difference in TARID expression in these HTR8/SVneo cells with CXCL3 overexpression or knockdown, which indicated that CXCL3 was regulated by TARID (Fig. 5i). These results demonstrated that TARID may affect the transmission of TNF signals, resulting in significant changes in CXCL3 expression.

### TARID activated the ERK/MAPK signaling pathway through CXCL3

**Fig. 6:**
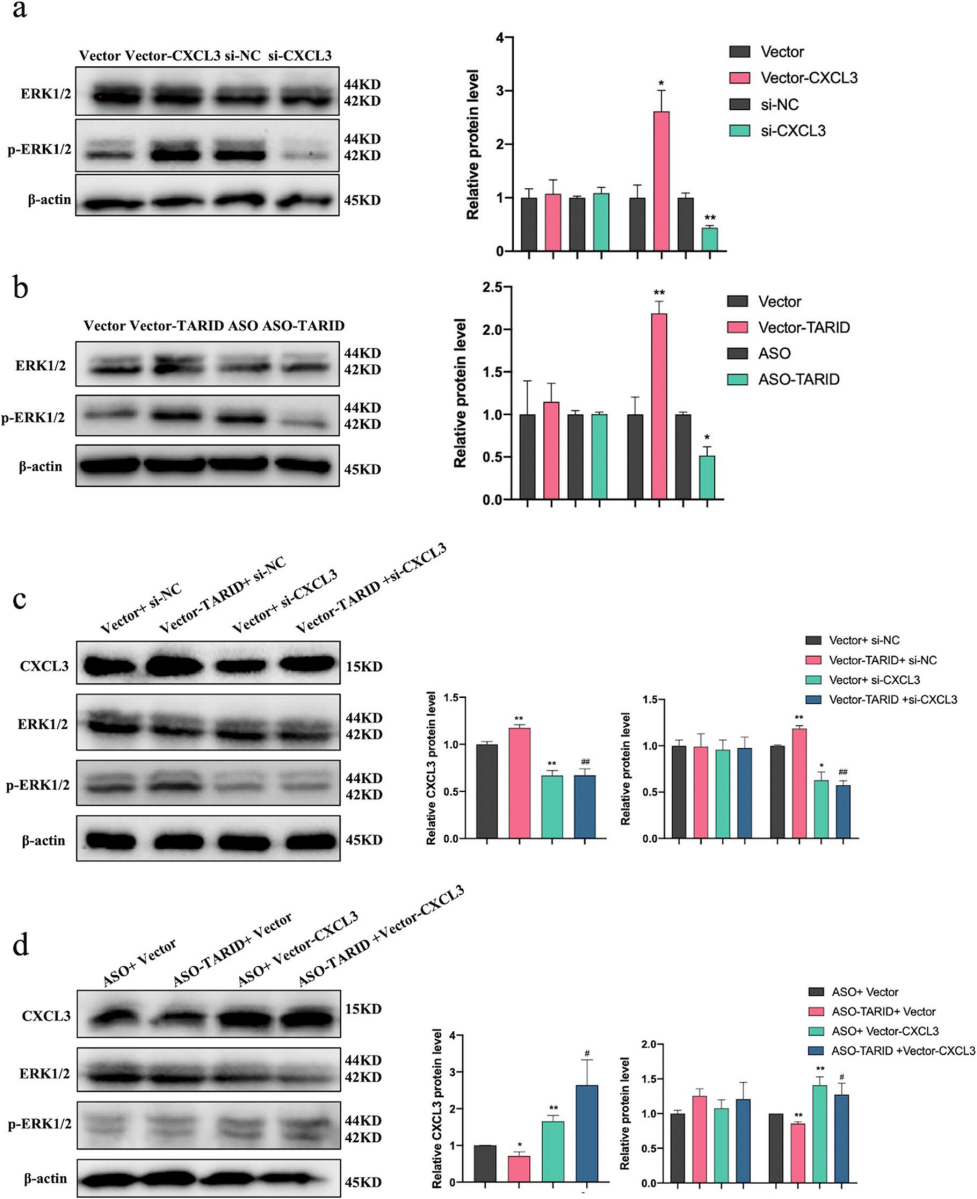
TARID activated the ERK/MAPK signaling pathway through CXCL3. **a** Results of western blotting showing the expression of ERK and p-ERK in HTR8/SVneo cells transfected with vector-CXCL3 or vector and si-CXCL3 or si-NC. **b** Results of western blotting showing the expression of ERK and p-ERK in HTR8/SVneo cells transfected with vector-TARID or vector and ASO-TARID or ASO. **c** Results of western blotting showing the expression of CXCL3, ERK and p-ERK in HTR8/SVneo cells co-transfected with vector-TARID and si-CXCL3. **d** Results of western blotting showing the expression of CXCL3, ERK and p-ERK in HTR8/SVneo cells co-transfected with ASO-TARID and vector-CXCL3. All experiments were conducted in triplicate; the values are shown as the mean ± SEM; vs. vector or ASO，**P < 0.01, *P < 0.05; vs. vector-TARID + si-NC or ASO-TARID + vector, ##*P* < 0.01, #*P* < 0.05

Our previous study showed that CXCL3 promoted the migration, invasion, proliferation, and tube formation of trophoblasts [17, 18]. In addition, CXCL3 affected the migration and invasion of airway smooth muscle cells, uterine cervical cancer cells and oral squamous cell carcinoma cells via mitogen-activated protein kinase (MAPK), with particular involvement of the extracellular signal-regulated kinase (ERK) pathway (ERK/MAPK) [19–21]. Next, we found that the phosphorylated ERK1/2 (p-ERK) level was increased when CXCL3 was overexpressed and that the p-ERK level was decreased when CXCL3 expression was knocked down, showing that CXCL3 affected the ERK/MAPK pathway in HTR8/SVneo cells (Fig. 6a). In addition, the expression of p-ERK was significantly higher when TARID was overexpressed, and that of p-ERK was decreased when TARID expression was knocked down in HTR8/SVneo cells (Fig. 6b).

Then, HTR8/SVneo cells were co-transfected vector-TARID and si-CXCL3 or ASO-TARID and vector-CXCL3, and changes to ERK/MAPK pathway activation were observed. A Western blot analysis showed that in vector-TARID and si-CXCL3 co-transfected HTR8/SVneo cells, p-ERK expression was significantly inhibited (Fig. 6c). CXCL3 overexpression reversed the p-ERK1/2 reduction caused by TARID knockdown (Fig. 6d). These results demonstrated that TARID may activate the ERK/MAPK signaling pathway through CXCL3.

### CXCL3 and the ERK/MAPK pathway activation was decreased in the placentas of PE patients

**Fig. 7:**
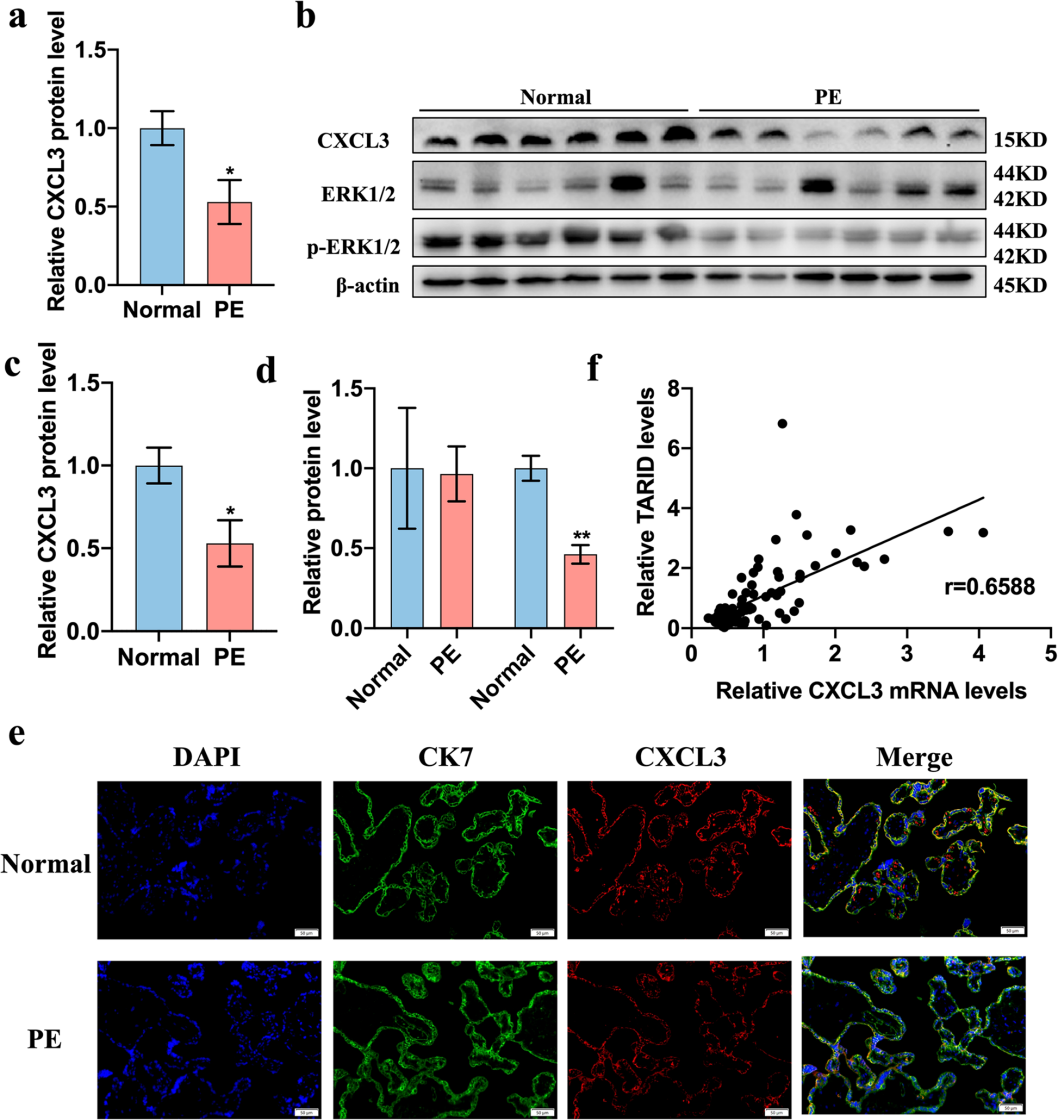
CXCL3 and MAPK/ERK pathway were aberrantly expressed in the placentas of patients with PE. **a** Results of real-time quantitative PCR showing the expression of CXCL3 in placentas (Normal = 40, PE = 43). **b-d** Results of western blotting showing the expression of CXCL3 in placentas. **e** Expression of CK7 and CXCL3 in placenta with the Immunofluorescence (200×). **f** Positive correlation (*r* = 0.6588) between CXCL3 mRNA and the relative expression of TARID in placentas (Normal = 40, PE = 43) (Pearson correlation analysis). All experiments were conducted in triplicate; the values are shown as the mean ± SEM; vs. normal，**P < 0.01, *P < 0.05

The levels of CXCL3, ERK1/2 and p-ERK were detected in the PE and normal placentas. The expression of CXCL3 was significantly decreased in the PE placentas at both the mRNA and protein levels (Fig. 7a-c). After adjusting for the differences in BMI and gestational age, CXCL3 expression remained significantly lower in the PE group (OR = 0.18) and was similar to the difference calculated before correction (OR = 0.13) (Table 3).

**Table 3:** The unadjusted and adjusted ORs of the relationship between preeclampsia and CXCL3 mRNA levels

	n	Unadjusted OR (95% CI)	*P* value	Adjusted OR (95% CI)	*P* value
Control	40	1	< 0.01	1	< 0.05
Preeclampsia	43	0.13(0.03–0.48)		0.18(0.04–0.89)	

Adjusted for BMI and gestational age, *ORs*, Odd ratios, *CIs* Confidence intervals

The protein level of p-ERK was decreased in the PE placentas compared with that in the normal control placentas (Fig. 7d), and the expression level of ERK in the PE placentas was not significantly different from that in the normal group (Fig. 7d). In addition, the expression level of CXCL3 mRNA in the placentas was positively correlated with the expression level of TARID (Fig. 7e, *r* = 0.6588).

## Discussion

PE is a common obstetric complication in pregnant women, and it seriously affects maternal and fetal health. The only way to prevent the progression of PE is to terminate the pregnancy [22, 23]. Hence, to the comprehensive identification of the pathological mechanisms of PE and discovery of new treatment methods are very important. The association between the abnormal expression of lncRNAs and PE has been studied in recent years [24]. Recently, some studies have shown that lncRNAs can regulate trophoblast invasion, differentiation of extravillous trophoblasts and spiral artery remodeling, which are key factors in PE development [5, 6, 9, 25, 26]. Due to the large number and complex functions of lncRNAs, much work remains to be done, especially mechanistic analyses. Therefore, we performed high-throughput sequencing to identify more PE-related lncRNAs and further study their functions and underlying mechanisms of action.

TARID was among the most differentially expressed lncRNAs, and TARID expression was significantly downregulated in 91% (39/43) of the PE patient placentas. A previous study has shown that TARID activated TCF21 expression by interacting with both the TCF21 promoter and GADD45A during tumor development [27]. TARID is broadly expressed in the placenta (RPKM 2.4), as indicated by RNA-seq of tissue samples from 95 human individuals representing 27 different tissues, suggesting that TARID may be an important molecule in the placenta [28]. However, the role played by TARID and its mechanism of action in other diseases and tissues remain to be discovered. After transcriptome sequencing of normal and PE placentas, we compared the results and found that the expression of TARID was significantly reduced and negatively correlated with blood pressure in PE patients, suggesting that it may be involved in an underlying mechanism of PE. Subsequently, we carried out studies with HTR-8/SVneo and JAR cells. Knocking down TARID expression inhibited trophoblast migration, invasion, and tube formation, while TARID overexpression had the opposite effect, suggesting that TARID may be involved in placental trophoblast engraftment and spiral artery remodeling.

To gain a better understanding of the mechanism by which TARID regulates trophoblast migration, invasion, and tube formation, mRNA-seq analysis was performed, and potential downstream effectors of TARID were identified. After overexpressing TARID, we found a significantly enriched pathway, the TNF signaling pathway, and a molecule that was significantly changed in this pathway, CXCL3. The TNF signaling pathway is involved in vital cellular functions, such as cell proliferation, morphogenesis, apoptosis, inflammation, and immune regulation [29]. For example, TNF-α influences hormone synthesis, placental architecture, and embryonic development in pregnancy [30]; Pyridostigmine ameliorated hypertension and other PE-like symptoms in rat models of PE by inhibiting the synthesis of TNF-α [31]. In the present study, CXCL3 was the most significantly altered effector in this pathway in HTR8/SVneo cells. Furthermore, we found no difference in TARID expression in THR8/SVneo cells with CXCL3 overexpressed or knocked down, indicating that CXCL3 was located downstream of TARID and was regulated by TARID. The expression of CXCL3, a member of the CXC-type chemokine subfamily, was significantly lower in the placentas of severe PE patients than that in patients with a normal pregnancy [17, 18]. Our previous research proved that exogenous recombinant CXCL3 promoted the migration and proliferation of trophoblasts and that endogenous CXCL3 was related to the migration, invasion, proliferation, and tube formation of trophoblasts, supporting our current research hypothesis suggesting that TARID acts through CXCL3 to affect trophoblast function [17, 18].

According to previous research, CXCL3-induced migration was dependent on the p38 and ERK1/2 MAPK pathways via CXCR1 and CXCR2 [19]. Moreover, the exogenous ERK1/2 blocker PD98059 attenuated CXCL3-induced cell proliferation and migration effects [20, 21]. We therefore hypothesized that TARID can regulate the ERK/MAPK pathway via CXCL3. Interestingly, we found increased p-ERK expression in HTR-8/SVneo cells overexpressing TARID and decreased p-ERK expression in HTR8/SVneo cells with TARID expression knocked down, suggesting that TARID regulated the ERK/MAPK pathway. The co-transfection experiments showed that knocking down CXCL3 expression in TARID-overexpressing HTR8/SVneo cells suppressed the increase in p-ERK expression and that overexpression of CXCL3 in TARID-knockdown HTR8/SVneo cells reversed the decrease in p-ERK expression. These results, as mentioned above, confirmed that TARID regulated the ERK/MAPK pathway via CXCL3. In addition, we verified this finding in the placenta. qPCR and Western blotting results showed that CXCL3 expression was decreased in the PE placenta sample, and p-ERK expression was also lower in the PE samples than that in the normal control samples. These experimental results suggested that TARID may affect trophoblastic infiltration and spiral artery remodeling through the CXCL3/ERK/MAPK pathway. Therefore, in the placenta of PE, the decrease of TARID and CXCL3 might eventually lead to abnormal placentation early in the first trimester and the reduced uteroplacental perfusion. Then decreased uteroplacental perfusion induces the placenta release excess placental antiangiogenic factor, soluble fms-like tyrosine kinase 1(sFlt1), which antagonizes proangiogenic factors, vascular endothelial growth factor (VEGF) and placental growth factor (PlGF), leading to endothelial dysfunction and systemic vascular dysfunction [32–34]. Meanwhile, ERK/MAPK participates in protecting against endothelial apoptosis [35], but it is down-regulation in PE and might be involved in endothelial dysfunction together with sFlt1, VEGF and PlGF.

Previous studies reported that GADD45A and TARID were specifically bound together [27]. GADD45A is a stress response protein related to DNA repair, the cell cycle, apoptosis, angiogenesis, and DNA demethylation [36–43]. PPARγ and GADD45A can pull each other down and colocalize in cells, indicating that GADD45A may regulate the transcriptional activities of PPARγ [44]. Furthermore, PPARγ2, an isoform of PPARγ, bound the CXCL3 promoter to regulate CXCL3 expression [45–47]. Considering these findings together, we speculate that TARID might regulate CXCL3 transcription via GADD45A and PPARγ. Therefore, in future research, we might focus more attention on the intricate regulatory mechanisms between TARID and CXCL3.

There are some limitations to our experiments. First, the gestational age between normal and PE patients was significantly different, which may have been a confounding factor when analyzing changes in placental TARID or CXCL3 mRNA expression. Indeed, finding an ideal control is preterm mother with no complications, but preterm birth is often the result of pregnancy complications or comorbidities, making identification of true controls unrealistic. Therefore, we took gestational age into account as a confounding factor in the logistic binary regression analysis, and the results showed that the effect of TARID were the same after correction, indicating that the effect of gestational age was limited. Second, our mechanistic study was performed only in vitro, and more in vivo studies, such as those performed with an established a mouse model of PE to verify the effect of TARID in vivo, have yet to be carried out. Third, the HTR8/SVneo and JAR cells were selected to demonstrate the role of TARID in biological behaviors of trophoblasts, which allowed the results to corroborate on multiple cells and increase the reliability of the results. However, JAR cell line is a choriocarcinoma cell line and a little different from normal trophoblasts. In the future, we could do further experiments with primary human placental trophoblasts, whose biological characteristics are closer to those in vivo*.* Fourth, the KEGG analysis suggest that TARID may be related to the apoptosis pathway, but we found no significant results in the apoptosis experiment of trophoblasts, which may be because TARID does not affect the main apoptotic molecules or is compensated by other molecules.

## Conclusions

In summary, we discovered that TARID is a novel PE-related lncRNA that promotes the migration, invasion, and tube formation of trophoblasts and participates in the occurrence and development of PE by regulating the CXCL3/ERK/MAPK pathway. These results may shed light on some of the mechanisms underlying spiral artery remodeling disorders and suggest that TARID may be a potential molecular target for the treatment of preeclampsia.

## Supplementary Information

**Additional file 1.**

## Data Availability

The datasets used and/or analyzed during the current study are available from the corresponding author on reasonable request.

## Abbreviations

PE: Preeclampsia
lncRNAs: Long noncoding RNAs
TARID: TCF21 antisense RNA inducing demethylation
ORs: Odds ratios
BMI: Body mass index
GO: Gene Ontology
KEGG: Kyoto Encyclopedia of Genes and Genomes
MAPK: Mitogen-activated protein kinase
ERK: Extracellular signal-regulated kinase
p-ERK: Phosphorylated ERK
sFlt1: Soluble fms-like tyrosine kinase 1
VEGF: Vascular endothelial growth factor
PlGF: Placental growth factor
