# Supplementary material for: The long noncoding RNA TARID regulates the CXCL3/ERK/MAPK pathway in trophoblasts and is associated with preeclampsia
Source: Reprod Biol Endocrinol. 2022 Nov 19;20:159. doi: 10.1186/s12958-022-01036-8 (PMC9675252; doi:10.1186/s12958-022-01036-8)
Supplement: Supplementary file 1 — Additional file 1. [file 12958_2022_1036_MOESM1_ESM.docx]

|  | **Maternal age (year)** | **BMI (kg/m2)** | **SBP (mm Hg)** | **DBP (mm Hg)** | **Gestational age (weeks)** | **Primipara (1)** | **Birth weight (g)** | **Birth length (cm)** | **Fetal sex (1= male)** | **Proteinuria (g/24h)** | **Platelet (10^9^/L)** | **ALT (U/L)** | **AST (U/L)** | **Total bilirubin (umol/L)** | **Albumin (g/L)** | **LDH (U/L)** | **BUN (mmol/L)** | **Serum creatinine (mmol/L)** |
| --- | --- | --- | --- | --- | --- | --- | --- | --- | --- | --- | --- | --- | --- | --- | --- | --- | --- | --- |
| Normal 1 | 32 | 20.34 | 120 | 79 | 37 | 1 | 2030 | 46 | 1 | - | 164 | 33 | 29 | 7.9 | 37 | 207 | 4.87 | 50 |
| Normal 2 | 39 | 25.15 | 103 | 61 | 39 | 0 | 3750 | 50 | 0 | - | 204 | 9 | 17 | 11.7 | 36.2 | 158 | 3.05 | 46 |
| Normal 3 | 35 | 25.91 | 100 | 71 | 39.14 | 0 | 3340 | 49 | 1 | - | 144 | 6 | 14 | 11.9 | 32.5 | 142 | 3.51 | 46 |
| Normal 4 | 32 | 26.9 | 127 | 74 | 38.86 | 1 | 2890 | 49 | 1 | - | 235 | 8 | 18 | 9.4 | 40.4 | 189 | 3.3 | 48 |
| Normal 5 | 27 | 27.16 | 115 | 78 | 37 | 1 | 3630 | 51 | 0 | - | 108 | 7 | 25 | 3.1 | 37.1 | 405 | 2.93 | 42 |
| PE 3 | 26 | 26.67 | 180 | 120 | 37 | 1 | 1360 | 38 | 0 | 0.14 | 197 | 12 | 21 | 2.5 | 38 | 512 | 5.01 | 72 |
| PE 4 | 29 | 27.69 | 173 | 111 | 32 | 0 | 1440 | 36 | 0 | 11.76 | 131 | 22 | 20 | 1 | 27.8 | 386 | 5.77 | 73 |
| PE 5 | 33 | 28.98 | 165 | 104 | 37.43 | 0 | 2440 | 46 | 0 | 1.36 | 292 | 18 | 27 | 3.9 | 32.1 | 392 | 4.23 | 49 |
| PE 1 | 35 | 30.41 | 183 | 88 | 35.14 | 1 | 3010 | 50 | 1 | 0.5 | 135 | 511 | 183 | 8.6 | 32.3 | 278 | 4.7 | 51 |
| PE 2 | 30 | 21.93 | 150 | 102 | 34.71 | 1 | 1970 | 42 | 1 | 1.22 | 81 | 12 | 15 | 8.1 | 34.2 | 215 | 9.05 | 88 |

Table S1. Clinical characteristics of five selected pairs of pregnant women in transcriptome sequence

Table S2

| Real-time PCR primer sequences | |
| --- | --- |
| TARID forward | 5′‐GACTCACAGATCCAAGAATCCCA‐3′ |
| TARID reverse | 5′‐CAGCAGTTTGGCAAGATGGAG‐3′ |
| TRAF1 forward | 5′‐CGGCGCCGAGATGGAG‐3′ |
| TRAF1 reverse | 5′‐GTGTGGTTCAACGTCACAGC‐3′ |
| LIF forward | 5′‐CTGCTGTTGGTTCTGCACTG‐3′ |
| LIF reverse | 5′‐GCCACATAGCTTGTCCAGGT‐3′ |
| CXCL2 forward | 5′‐AGATCAATGTGACGGCAGGG‐3′ |
| CXCL2 reverse | 5′‐TGCTCTAACACAGAGGGAAACA‐3′ |
| CXCL3 forward | 5′‐TGAATGTAAGGTCCCCCGGA‐3′ |
| CXCL3 reverse | 5′‐CACCCTGCAGGAAGTGTCAA‐3′ |
| IL6 forward | 5′‐TTCGGTCCAGTTGCCTTCTC‐3′ |
| IL6 reverse | 5′‐TGTTTTCTGCCAGTGCCTCT‐3′ |
| PTGS2 forward | 5′‐AGTCCCTGAGCATCTACGGT‐3′ |
| PTGS2 reverse | 5′‐GCCTGCTTGTCTGGAACAAC‐3′ |
| EDN1 forward | 5′‐GCTGCCTTTTCTCCCCGTTA‐3′ |
| EDN1 reverse | 5′‐CTCCTTGGCAAGCCACAAAC‐3′ |
| FOS forward | 5′‐CCGAGCTGGTGCATTACAGA‐3′ |
| FOS reverse | 5′‐ACACACTCCATGCGTTTTGC‐3′ |
| β‐actin forward | 5′‐CATGTACGTTGCTATCCAGGC‐3′ |
| β‐actin reverse | 5′‐CTCCTTAATGTCACGCACGAT‐3′ |
| U6 forward | 5′‐CTCGCTTCGGCAGCACA‐3′ |
| U6 reverse | 5′‐AACGCTTCACGAATTTGCGT‐3′ |
| RNAi sequence | |
| TARID ASO sequence (human) | GCTGTTCTCATAACTGATGT |
| CXCL3 siRNA sequence (human) | GGAAGAAAGCTTGTCTCAA |
